# Skin pigmentation gradually decreases with age under non‐ultraviolet exposure conditions

**DOI:** 10.1002/ski2.193

**Published:** 2023-02-02

**Authors:** Jing Chen, Li Lei, Ling Jiang, Yibo Hu, Jinhua Huang, Qinghai Zeng

**Affiliations:** ^1^ Department of Dermatology Third Xiangya Hospital Central South University Changsha China

## Abstract

In non‐sun exposure conditions, skin pigmentation gradually decreases with age. Sun exposure on the other hand increases skin pigmentation. In addition, the skin of the elderly is more prone to tanning than youthful skin.

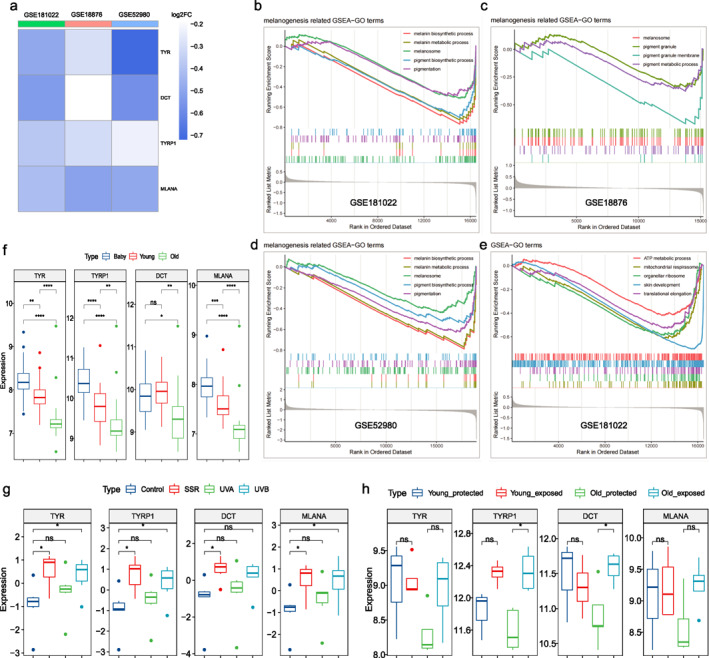

## ETHICS STATEMENT

Not applicable.

Dear Editor,

Ultraviolet (UV) is the major extrinsic driver of skin pigmentation.^1^, ^2^, ^3^ However, little is known regarding skin melanogenesis in the absence of UV exposure. Our objective was to explore the changes in melanogenesis at different ages in the absence of UV exposure based on transcriptomic data.

The transcriptome array results of skin tissues from different age groups under non‐UV exposure conditions were retrieved from the GEO database. Three RNA‐array datasets (GSE181022,^4^ GSE18876,^5^ and GSE52980^6^) of various ages were included, of which one (GSE181022), also included babies. In this study, baby is defined as a person under 2 years old, young is defined as a person between 18 and 40 years old, and old is defined as a person over 55 years old. Data was analyzed by R (version 4.0.3). The differentially expressed genes related to melanogenesis were identified using the limma R package with log2 fold change as the criterion. Heatmap R package was used to construct a heatmap. Gene Set Enrichment Analysis (GSEA) was performed using clusterProfiler R package to analyze the changes in Gene Ontology. Wilcox test or *t* test was used to compare the groups. In addition, RNA‐array datasets of skin (GSE21429^7^ and GSE52980) irradiated with different sources and different doses of UV were also analyzed. Ethnicity and skin phototype are important factors affecting skin melanogenesis. Unfortunately, not every database set out data on race or skin phototype. Therefore, we were unable to distinguish differences in melanogenesis at different skin phototypes.

**FIGURE 1:**
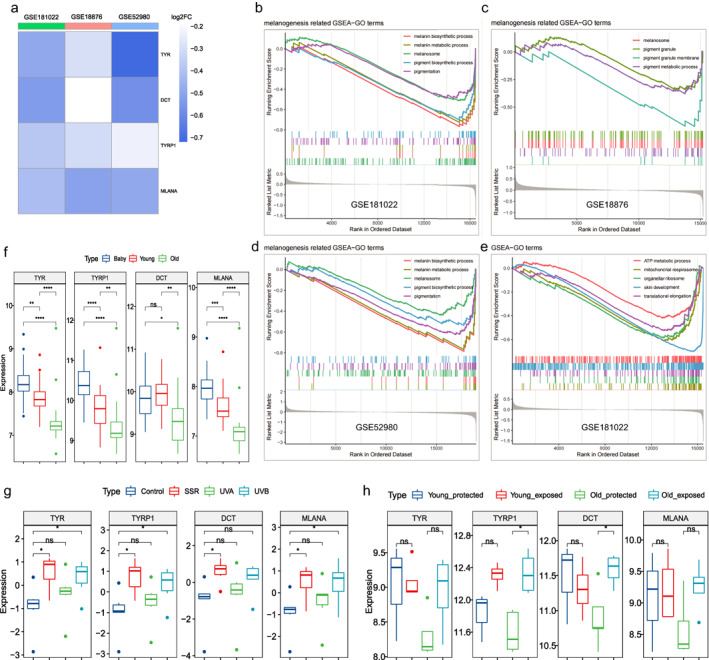
Skin pigmentation gradually decreases with age under non‐UV exposure conditions. The log2 fold change (old vs. young) of melanogenesis‐related genes in datasets of (a) GSE181022 (baby: *n* = 27, 30.7–89.1 week; young: *n* = 25, 20–24 years; old: *n* = 18, 60–64 years), GSE18876 (young: *n* = 25, 19–39 years; old: *n* = 44, 55–86 years) and GSE52980 (young: *n* = 3, under 35 years old; old: *n* = 5, over 60 years old). The full and ordered gene list of differentially expressed genes (DEGs) was analyzed by Gene Set Enrichment Analysis (GSEA). Melanogenesis‐related Gene Ontology (GO) terms of GSEA (old vs. young) in (b) GSE181022, (c) GSE18876 and (d) GSE52980. (e) Cellular activity‐related GO terms of GSEA (old vs. young) in GSE181022. (f) The expression of melanogenesis‐related genes in the skin of baby, young, and elderly individuals. (g) The effect of different sources of UV on skin melanogenesis (h) and the responses of young and old skin to UV. *p < 0.05, **p < 0.01, ****p < 0.0001, ns: *p* > 0.05.

Surprisingly, analysis of the GSE181022, GSE18876 and GSE52980 datasets indicated that the expression of melanogenesis‐related genes (TYR, TYRP1, DCT and MLANA) was significantly lower in the elderly skin compared to that of younger individuals (Figure 1a). GSEA further showed that multiple functions related to melanogenesis (Figure 1b–d), cellular activities and skin development (Figure 1e) were significantly inhibited in the old skin samples, suggesting that the skin functions generally decline with age. And the decrease in melanogenesis may be secondary due to the degenerative changes in melanocytes function, which requires further verification. Interestingly, the results from GSE181022 datasets revealed that the expression of melanogenesis‐related genes showed a decreasing trend in babies, young and the elderly (Figure 1f). Compared to the babies, the skin of young individuals also showed a significant reduction in the melanogenesis‐related genes (Figure 1f), suggesting that while skin functions evolve after birth, melanogenesis in young individuals is lower than that in babies. This can be attributed to the hypothesis that complex amniotic fluid surrounds the skin of the foetus induces melanogenesis. However, the absence of strong stimuli (including amniotic fluid and UV) after birth likely does not effectively stimulate melanogenesis.

We further analyzed the effects of different sources of UV on skin melanogenesis with RNA‐array datasets of skin (GSE21429), and found that solar‐simulated radiation (SSR) and ultraviolet B (UVB) could significantly promote melanogenesis (Figure 1g), while UVA had no significant effect. Consistent with previous report,^7^ SSR and UVB have a greater effect on melanogenesis than UVA. Furthermore, we compared the responses of young and old skin to UV in GSE52980 datasets, and found that UV‐induced expression of melanogenesis‐related genes was stronger in the latter (Figure 1h). Compared to younger skin, that of the elderly is more likely to be tanned, resulting in more pigmentation spots in the facial skin.

In conclusion, in non‐sun exposure conditions, skin pigmentation gradually decreases with age. Sun exposure on the other hand increases skin pigmentation. In addition, the skin of the elderly is more prone to tanning than youthful skin. Our study is only based on transcriptomic data and will have to be elucidated with functional studies.

## CONFLICT OF INTEREST

The authors declared that they have no conflicts of interest to this work.

## FUNDING INFORMATION

National Natural Science Foundation of China, Grant/Award Numbers: 82073421, 82073420.

## AUTHOR CONTRIBUTIONS

**Jing Chen**: Project administration (Lead); Writing – review & editing (Lead). **Li Lei**: Project administration (Lead); Software (Equal); Writing – original draft (Equal); Writing – review & editing (Lead). **Ling Jiang**: Conceptualization (Equal); Data curation (Equal); Formal analysis (Equal). **Yibo Hu**: Investigation (Equal); Methodology (Equal); Resources (Equal); Software (Equal). **Jinhua Huang**: Project administration (Equal); Supervision (Equal). **Qinghai Zeng**: Funding acquisition (Equal); Project administration (Equal); Supervision (Equal); Writing – review & editing (Equal).

## Data Availability

The data that support the findings of this study are openly available in GSE181022, GSE18876, GSE52980, GSE21429, and GSE22083 from GEO database.
